# ST32da, a Novel *Salvia miltiorrhiza*-Derived ATF3 Inducer, Alleviates Obesity-Related Diabetic Nephropathy in Mouse Models

**DOI:** 10.3390/cells14231893

**Published:** 2025-11-28

**Authors:** Hsi-Hsien Chen, Tzu-Ling Tseng, Hsiao-Fen Li, Ya-Ting Hsieh, Tu Tuan Tran, Yueh-Lin Wu, Heng Lin

**Affiliations:** 1 Division of Nephrology, Department of Internal Medicine, Taipei Medical University Hospital, No. 252, Wuxing St., Xinyi Dist., Taipei 110, Taiwan; d102093015@tmu.edu.tw; 2 Division of Nephrology, Department of Internal Medicine, School of Medicine, College of Medicine, Taipei Medical University, No. 250, Wuxing St., Xinyi Dist., Taipei 110, Taiwan; 3 TMU Research Center of Urology and Kidney, Taipei Medical University, Taipei 110, Taiwan; 4 Division of Endocrinology and Metabolism, Department of Internal Medicine, Taipei Medical University Hospital, No. 252, Wuxing St., Xinyi Dist., Taipei 110, Taiwan; 5 CardioVascular Research Center, Buddhist Tzu Chi General Hospital, No. 707, Zhongyang Rd., Sec. 3, Hualien 97004, Taiwan; lingtseng@tzuchi.com.tw; 6 Department of Medical Research, Hualien Tzu Chi Hospital, Buddhist Tzu Chi Medical Foundation, Hualien 97004, Taiwan; 7 Department of Basic Medical Sciences, College of Nursing, Tzu Chi University, Hualien 97004, Taiwan; 8 Department of Physiology, School of Medicine, College of Medicine, Taipei Medical University, Taipei 110, Taiwan; bubble0728@tmu.edu.tw (H.-F.L.);; 9 Department of Internal Medicine, Thai Nguyen University of Medicine and Pharmacy, Thai Nguyen 241-17, Vietnam; trantuantu@tump.edu.vn; 10 Department of Nephro-Urology and Dialysis, Thai Nguyen National Hospital, Thai Nguyen 241-24, Vietnam; 11 Division of Nephrology, Department of Internal Medicine, Wan Fang Hospital, Taipei Medical University, No. 111, Sec. 3, Xinglong Rd., Taipei 11696, Taiwan

**Keywords:** ST32da, ATF3, diabetic nephropathy, renal inflammation, HDAC2, lipotoxicity

## Abstract

**Highlights:**

**What are the main findings?**

- ST32da enhances ATF3/HDAC2 recruitment to inhibit NF-κB–IL-6 signaling.
- ST32da mitigates podocyte injury and kidney lipotoxicity in diabetic mice.

**What is the implication of the main finding?**

- ST32da show potential for use in treating diabetic nephropathy.

**Abstract:**

It is necessary to find novel therapeutic strategies for obesity-related diabetic nephropathy (DN) that target both metabolic dysfunction and renal inflammation. ST32da derived from *Salvia miltiorrhiza* (a well-recognized Traditional Chinese Medicine) induces activating transcription factor 3 (ATF3), a negative regulator of inflammation and metabolic stress. However, the effects of ST32da on obesity-related DN remain underexplored. We investigated the therapeutic potential of ST32da, a synthetic ATF3 inducer derived from *Salvia miltiorrhiza*, in mitigating obesity-related DN in both in vivo and in vitro models. The Nephroseq database analysis was performed to explore the relationship between Atf3 expression and DN progression. ST32da was administered to db/db knockout and DBA mice to establish obesity-related DN models, and a high-fat diet (HFD)-induced mouse model of obesity-related DN was used to investigate the effects of Atf3 knockout. Molecular and biochemical analyses were conducted in cultured mesangial cells to elucidate the underlying mechanisms. ATF3 deficiency worsened obesity-related DN, increasing glomerular fibrosis, mortality, and inflammation. ST32da restored ATF3 levels and reduced renal injury, glomerular expansion, and pro-inflammatory cytokine expression (e.g., IL-6, TGFβ, TNFα). ST32da-treated mice exhibited reduced hepatic lipid accumulation and improved serum lipid profiles. In mesangial cells, ST32da localized to the cytoplasm and increased ATF3 activity, which suppressed RARRES1 expression and cytokine signaling. Mechanistically, ATF3 interacted with HDAC2 to repress NF-κB—dependent inflammatory gene expression. The findings suggest ST32da is a promising therapeutic candidate for obesity-related DN and associated metabolic disturbances, acting through ATF3 induction to suppress renal inflammation, lipotoxicity, and fibrosis.

## 1. Introduction

Obesity, defined as a body mass index of ≥30 kg/m^2^, has surged to epidemic levels and is a substantial global health issue closely associated with chronic conditions, such as type 2 diabetes mellitus (T2DM), hypertension, non-alcoholic fatty liver disease, and cardiovascular diseases. Diabetic nephropathy (DN) is a severe microvascular complication of T2DM and a leading cause of end-stage renal disease, which currently lacks effective therapeutic strategies. DN results from prolonged hyperglycemia; it leads to kidney injury and is characterized by chronic inflammation, hemodynamic changes, and metabolic dysfunction [1]. Initial DN pathology includes podocyte damage, shedding, apoptosis, and compensatory hypertrophy and fusion in surviving podocytes [2]. Podocyte effacement, apoptosis, and reduced podocyte numbers per glomerulus have been identified as the key predictors of DN progression [3,4]; however, the precise underlying mechanism of DN is elusive. Therefore, further investigation into the molecular mechanisms that link chronic inflammation and metabolic dysfunction in DN is needed to inform more effective therapies and to prevent progression to end-stage renal disease.

Factors such as glycolipid toxicity, angiotensin II, and advanced glycation end products trigger podocyte apoptosis via various proapoptotic pathways, leading to DN [5]. The interleukin-6 (IL-6) signaling pathway affects renal-resident cells, including podocytes, mesangial cells, endothelial cells, and tubular epithelial cells, and has been implicated in several renal diseases, including DN. Moreover, it has been speculated that local activation of IL-6 classic and trans-signaling pathways contributes to renal autoimmune and inflammatory diseases, potentially causing apoptosis in kidney cells [6]. Hyperglycemia induces IL-6 upregulation, promoting podocyte hypertrophy, apoptosis, and cytoskeletal disruption [7,8]. Blocking IL-6 and its receptors reduces renal injury in diabetic rodents [9,10], highlighting IL-6 as a therapeutic target.

In parallel with inflammation, the direct induction of pro-apoptotic signaling pathways also contributes significantly to podocyte loss. Recent studies have indicated that retinoic acid RA receptor responder protein 1 (RARRES1) expression correlates with primary glomerular disease progression, suggesting its association with kidney disease [11,12]. RARRES1, a type I transmembrane protein originally identified as a retinoic acid-responsive gene [13], is upregulated in podocytes during DN, and it promotes apoptosis by inhibiting RIO kinase 1 (RIOK1) and activating p53 [12]. In vivo, its overexpression exacerbates glomerular damage and albuminuria, while a cleavage-resistant mutant shows no effect [11]. Additionally, soluble RARRES1 contributes to tubular injury and lipid accumulation. These findings suggest that targeting RARRES1 may offer therapeutic potential in DN.

Traditional Chinese Medicine has gained attention as a complementary approach for managing chronic diseases, including DN. *Salvia miltiorrhiza* Bunge, a widely used Traditional Chinese Medicine herb, is known for its antioxidant, anti-inflammatory, and microcirculatory benefits [14,15,16]. Prior studies have demonstrated its potential in improving glucose and lipid metabolism [17,18] and in slowing kidney disease progression [19,20].

In our previous study, we identified ST32da, a synthetic compound derived from *Salvia miltiorrhiza*, as an inducer of activating transcription factor 3 (ATF3) [21]. ST32da downregulated adipokine genes and promoted adipocyte browning by inhibiting the carbohydrate-responsive element-binding protein–stearoyl-coA desaturase-1 axis [21]. Additionally, ATF3 was shown to directly interact with histone deacetylase 1 (HDAC1), facilitating the translocation of HDAC1 to the ATF/NF-κB regulatory elements on *IL6* promoters. This interaction promotes HDAC1-mediated histone deacetylation, resulting in chromatin compaction and NF-κB binding disruption, thereby downregulating pro-inflammatory IL6 expression and reducing apoptosis during ischemic reperfusion injury [22]. Based on this evidence, we hypothesized that ST32da may protect against DN by modulating ATF3-mediated suppression of IL-6 signaling and downstream podocyte injury pathways.

To test this hypothesis, in this study, we evaluated the effect of ST32da on glomerular podocytes in DN in vitro using mouse podocyte clone 5 (MPC5) and mesangial cells, and in vivo using diabetic mouse models. The findings of this study will inform future research on epigenetic therapies for DN and support the development of ST32da as a potential therapeutic agent

## 2. Materials and Methods

### 2.1. Animal Studies and ST32da

Eight-week-old C57BL/6, Atf3^−/−^, DBA/2, and db/db male mice were used in this study. Atf3^−/−^ mice were provided by Dr. Tsonwin Hai as in our previous study [23]. To induce T2DM, 5-week-old C57BL/6J and Atf3^−/−^ mice were fed a 60 kcal% high-fat diet (HFD) for four weeks, followed by intraperitoneal (i.p.) injections of streptozotocin (STZ, 60 mg/kg) three times a week. To monitor the effects of ST32da, 8-week-old DBA mice were fed an HFD for 4 weeks, followed by a single STZ injection (65 mg/kg) and subsequent treatment with or without i.p. injection of ST32da (5 mg/kg twice per week) for six weeks. Additionally, 5-week-old db/db mice were fed a chow diet (normal diet, ND; 4% kcal from fat) for 2 weeks, then treated with ST32da (i.p.; 5 mg/kg twice per week) for 12 weeks. Body weights were monitored weekly. Sixteen weeks post-ST32da treatment, final body weight, kidney tissue, insulin sensitivity, glucose tolerance, and urine biochemical parameters were measured. Blood samples were centrifuged at 4 °C at 6000× *g* for 3 min, and kidney tissues were fixed in 4% paraformaldehyde for pathological analyses. All animal experimental procedures were performed following the protocols approved by the Institutional Animal Care and Utilization Committee of Taipei Medical University, Taipei, Taiwan. ST32da was generously provided by our collaborating laboratory, under the leadership of Professor Wei-Jan Huang. The synthesis protocol for ST32da has been described in previous studies [21,24], and is briefly summarized in Figure S1. To ensure rigorous quality control of ST32da samples, we employed nuclear magnetic resonance spectroscopy analysis Figures S2 and S3.

### 2.2. Cell Culture

Rat mesangial cells (RMC) were obtained from BCRC (#60415; Hsinchu, Taiwan) and cultured following the manufacturer’s instructions. Podocytes were then exposed to normal glucose (5.5 mM) and high glucose (25 mM) with 1% fetal bovine serum, with or without ST32da, for 2, 4, 6, and 8 h. Immortalized mouse podocyte clone 5 (MCP-5) cells [25] obtained from Moin A Saleem (University of Bristol, UK) were maintained in RPMI 1640 medium (Gibco; Thermo Fisher Scientific, Inc., Waltham, MA, USA) with 10% fetal bovine serum, 1% penicillin-streptomycin (Gibco), and 10 U/mL recombinant mouse interferon-γ (Sigma-Aldrich, St. Louis, MO, USA) at 33 °C (5% CO_2_, 90% humidity). Before experiments, cells were transferred to a 37 °C incubator without interferon-γ for at least 10 days to ensure full differentiation. Each experimental condition was repeated at least three times.

For IL-6 stimulation, podocytes were treated with 0.1 or 1 ng/mL IL-6, a range that activates the canonical IL-6 downstream pathway. These concentrations are sufficient to induce caspase-3 activation while avoiding supraphysiologic exposure, and are consistent with doses (≤10 ng/mL) commonly used in previous podocyte studies [26].

### 2.3. Histopathology

Kidney samples were fixed in 4% paraformaldehyde (Thermo Fisher Scientific, Inc.), embedded in paraffin, and sectioned to 4 μm thickness. Periodic acid-Schiff and Masson’s trichrome staining were used to examine kidney histology.

### 2.4. Transmission Electron Microscopy

Tissues were fixed in 2.5% glutaraldehyde with 0.1 M sodium cacodylate (pH 7.4) for 72 h at 4 °C, followed by incubation with 2% osmium tetroxide and 0.1 M sodium cacodylate (pH 7.4) for 1 h at room temperature. Subsequently, ultrathin sections were stained with lead citrate and uranyl acetate and viewed using a Hitachi HT-7700 microscope. (Hitachi Ltd’s, Tokyo, Japan)

### 2.5. Urine and Blood Biochemical Assays

Blood samples (500 µL) collected from the tail vein were centrifuged (6000× *g*; 3 min, 4 °C) to separate serum samples. Subsequently, serum biochemical parameters, including blood urea nitrogen (BUN), creatinine, glucose, and triglyceride, were measured within 24 h using Spotchem EZ SP 4430 (ARKRAY, Kyoto, Japan). Additionally, urine samples were collected and the levels of albumin and creatinine were quantified using E-90AL (Immunology Consultants Laboratory, Portland, OR, USA) and QuantiChrom Creatinine Assay Kit (DICT-500; Bioassay Systems, Hayward, CA, USA), respectively, and expressed as the albumin-to-creatinine ratio.

### 2.6. Real-Time PCR

Total RNA was extracted from cultured cells using TRIzol reagent (Invitrogen), reverse-transcribed to cDNA using the iScript cDNA Synthesis Kit (Bio-Rad, Hercules, CA, USA), and quantified using real-time quantitative PCR (RT-qPCR). The PCR amplification was carried out on ABI StepOnePlus Real-Time PCR System (Applied Biosystems, Grand Island, NY, USA) with SYBR Green (Bio-Rad). Primer sequences used for RT-qPCR are shown in Table 1.

### 2.7. Immunoprecipitation

The immunoprecipitation assay was conducted using an Abcam immunoprecipitation kit (ab206996; Cambridge, UK) per the manufacturer’s instructions. Briefly, cells were suspended and incubated in immunoprecipitation buffer at 4 °C. Subsequently, cells were incubated for 4 h with anti-ATF3 antibody (ab254268; Abcam, Cambridge, UK) and normal rabbit immunoglobulin G (negative control; ab172730; Abcam), followed by precipitation with Protein A/G Sepharose beads and overnight incubation at 4 °C. The immune complexes were washed thrice, eluted, and analyzed using Western blotting to assess protein expression.

### 2.8. Western Blotting

Kidney and cell extracts were separated on SDS-PAGE and analyzed by Western blotting using an ECL kit (Thermo Fisher Scientific, Inc.) following the kit instructions. The antibodies used included anti-ATF3 (#NBP-1-85816) from Novus Biological (Centennial, CO, USA), anti-HDAC1 (#5356), anti-HDAC 2 (#57156), anti-Phospho-NF-κB p65 (#3033), anti-Cleaved caspase-3 (#9664), and anti-Lamin A/C (#4777) from Cell Signaling Technology (Danvers, MA, USA); and anti-RARRES1 (#MA5-26246) and anti-β-actin (#MA5-15739) from Invitrogen (Thermo Fisher Scientific, Inc.).

### 2.9. Statistical Analyses

Results are presented as means ± standard error of means (SEM) from a minimum of three experiments. Statistical significance was assessed using Student’s *t*-test and two-way ANOVA, followed by Tukey’s test for in vivo experiments, with *p* < 0.05 deemed significant.

## 3. Results

### 3.1. Reduced ATF3 Expression in Human DN Datasets

**Figure 1:**
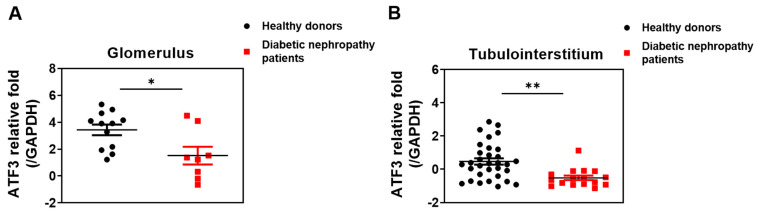
Analysis of ATF3 expression in normal individuals and patients with type 2 diabetes using Nephroseq database. (**A**) Glomerulus; (**B**) Tubulointersititum. Data represent mean ± SEM; * *p* < 0.05, ** *p* < 0.01.

ATF3 has been reported to protect against acute kidney injury by modulating inflammatory responses and apoptosis [22,27]. However, its role in chronic kidney diseases, such as DN, remains poorly defined. Animal models of proteinuric and DN (including lipopolysaccharide-treated C57BL/6 mice and db/db mice) have shown upregulation of ATF3 expression in podocytes under stress conditions, such as hyperglycemia and endotoxemia [28]. Moreover, gene polymorphism studies have implicated ATF3 in human obesity [29], suggesting broader metabolic relevance. To specifically investigate the involvement of ATF3 in DN, we analyzed publicly available human kidney transcriptomic data from the Nephroseq database (University of Michigan, https://www.nephroseq.org/resource/login.html, accessed on 20 October 2025), which encompasses extensive sequencing datasets from patients with various kidney pathologies and healthy controls. Our analysis revealed a significant downregulation of ATF3 expression in both the glomerular and tubulointerstitial compartments of kidneys from patients with DN (Figure 1A,B), suggesting that decreased ATF3 levels may be associated with DN progression.

### 3.2. Loss of Atf3 in Mice Aggravated DN

**Figure 2:**
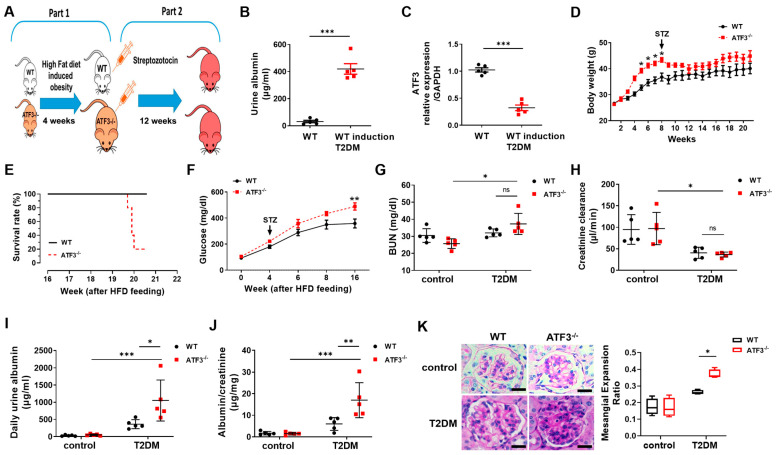
Loss of ATF3 in mice with aggravated high-fat diet (HFD)-induced diabetic nephropathy after STZ treatment. (**A**) Experimental procedure for inducing diabetic nephropathy in wild-type (WT) and *Atf3* knockout (*Atf3^−/−^*) mice. *Atf3^−/−^* mice and their WT littermates were fed an HFD for 4 weeks, followed by STZ infusion and maintenance with HFD for 12 weeks. (**B**) Urinary albumin levels in mice. (**C**) *Atf3* mRNA levels. (**D**) Body weight. (**E**) Survival rate. (**F**) Serum glucose levels. (**G**) Serum BUN levels. (**H**) Serum creatinine clearance. (**I**) Daily urinary albumin. (**J**) Urinary albumin/creatinine ratio. (**K**) Representative images of kidney tissue sections stained with Periodic Acid-Schiff (PAS) (left panel). Semi-quantitative analysis of the mesangial expansion ratio (right panel). Measurements were performed on ≥20 glomeruli per animal. Scale bars = 50 µm. Dot plots show individual values with bars representing the mean ± SEM; *n* = 5 for both WT and *Atf3^−/−^*; * *p* < 0.05, ** *p* < 0.01, *** *p* < 0.005.

To further explore the relationship between ATF3 and DN, we used Atf3^−/−^ mice and confirmed complete ablation of Atf3 expression in their renal tissue using quantitative RT-PCR (Figure S4A) and Western blot analysis (Figure S4B). Both wild-type (WT) and Atf3^−/−^ mice were treated with HFD and STZ to induce DN (Figure 2A). An assessment of the DN phenotypes revealed higher urine albumin levels in HFD/STZ-treated mice than those in placebo-treated mice (Figure 2B), with significantly reduced Atf3 mRNA expression in Atf3^−/−^ mice (Figure 2C). Furthermore, before STZ treatment, Atf3^−/−^ mice had significantly higher body weight than WT mice, consistent with clinical observations of weight loss in advanced DN [30]. However, the differences were not observed after STZ treatment (Figure 2D), suggesting that Atf3 plays an important pathophysiological role in the progression of DN.

Atf3^−/−^ mice exhibited a significantly reduced survival rate of 20%, whereas that of the WT mice remained at nearly 100% (Figure 2E). After STZ treatment, both groups had elevated serum glucose, with Atf3^−/−^ mice exhibiting a significantly higher level (Figure 2F). Furthermore, after the induction of DN, Atf3^−/−^ mice exhibited notably higher serum BUN levels (Figure 2G), 24 h urine albumin quantities (Figure 2I), and urine albumin-to-creatinine ratios (Figure 2J), but similar creatinine clearance levels (Figure 2H) when compared to their WT counterparts. These changes were accompanied by advanced mesangial expansion, a critical pathological feature of DN, in Atf3^−/−^ mice (Figure 2K). These findings suggest that the absence of Atf3 exacerbates the severity of DN.

### 3.3. Administration of ST32da, an Atf3 Inducer, Ameliorated Obesity-Induced DN in db/db Mice

We first confirmed that ST32da was able to induce ATF3 expression in vitro using HK-2 human proximal tubular epithelial cells. Both quantitative RT-PCR and Western blot analyses demonstrated a dose-dependent increase in ATF3 mRNA and protein levels (Figure S5A,B), confirming the biological activity of ST32da in activating ATF3 in vitro. Within the range of concentrations investigated, only the 50 µM concentration demonstrated a statistically significant elevation in both transcript and protein levels relative to the vehicle control. This effective dose subsequently informed the design of our in vivo experiments.

**Figure 3:**
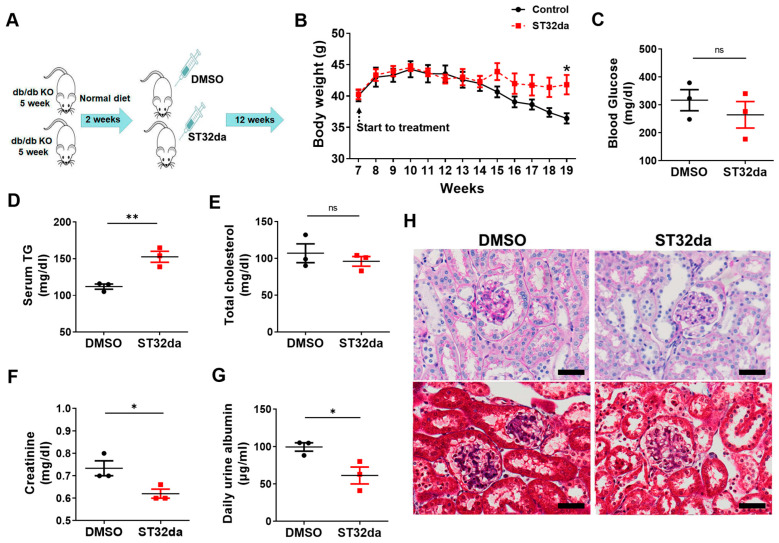
Effect of ATF3 inducer, ST32da, on db/db mice. (**A**) Schematic representation of the experimental schedule. Briefly, db/db mice were fed a normal chow diet for 2 weeks, followed by i.p. infusion with ST32da (5 mg/kg twice per week) for 12 weeks. Effects on (**B**) body weight, (**C**) serum glucose, (**D**) triglyceride (TG), (**E**) cholesterol, (**F**) creatinine, and (**G**) urine albumin measured at the end of the experiment. (**H**) Representative images of kidney tissue sections stained with PAS and Masson’s trichrome. * *p* < 0.05, ** *p* < 0.01.

Next, we investigated the effects of ST32da, on obesity-exacerbated DN using db/db mice, a monogenic model of T2DM with insulin resistance. The mice were treated with ST32da or placebo for 20 weeks (Figure 3A). ST32da-treated mice showed increased body weights compared with the placebo-treated mice (Figure 3B). However, serum glucose and cholesterol levels did not differ between the ST32da-treated and untreated groups (Figure 3C,E). In contrast, triglyceride levels were elevated in the ST32da group compared with those in the placebo group (Figure 3D). Furthermore, ST32da-treated mice exhibited significantly reduced serum creatinine and daily urine albumin excretion (Figure 3F,G). Histological analysis revealed preserved outer cortical glomeruli size and configuration, typical Bowman’s capsule caliber, and absence of epithelial cell proliferation in ST32da-treated mice (Figure 3H). Additionally, mesangial expansion was significantly less pronounced in the ST32da group compared with that in the placebo group (Figure 3H). These findings indicate that ST32da treatment effectively attenuates obesity-induced DN progression in the db/db mouse model.

### 3.4. ST32da, Ameliorated Glomerular Structural Changes, Podocyte Loss, and Fibrosis in STZ-HFD Treated DBA/2 Mice

**Figure 4:**
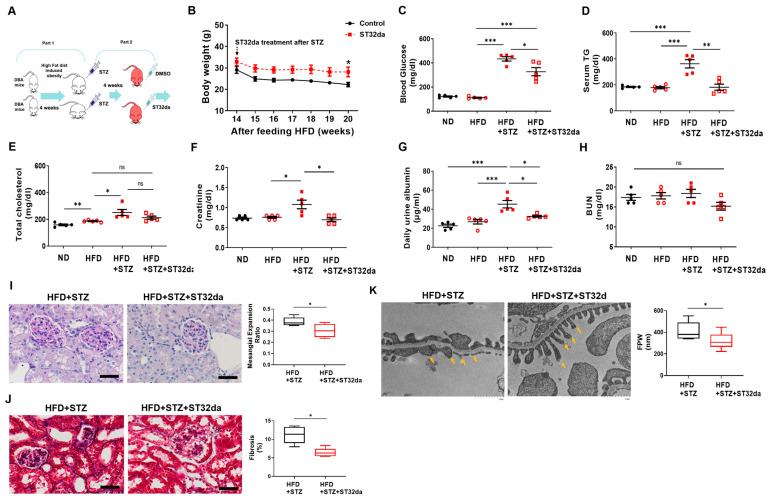
Effects of ATF3 inducer, ST32da, in STZ-HFD-treated DBA mice. (**A**) Schematic representation of the experimental schedule. Briefly, after 4 weeks of high-fat diet feeding, mice were injected with STZ (75 mg/kg) via the tail vein 3 times over one week. Subsequently, ST32da (5 mg/kg twice per week) was infused for six weeks. Effects on (**B**) body weight, (**C**) serum glucose, (**D**) triglyceride (TG), (**E**) cholesterol, (**F**) BUN, (**G**) creatinine, and (**H**) urine albumin assessed at the end of the experiment. (**I**) Representative images of kidney tissue sections stained with PAS (left panel). Semi-quantitative analysis of the mesangial expansion ratio (right panel). Measurements were performed on ≥20 glomeruli per animal (*n* = 5 per group). Scale bars = 50 µm. (**J**) Representative images of kidney tissue sections stained with Masson’s trichrome (left panel). Semi-quantitative analysis of fibrotic area (right panel). Measurements were obtained from ≥10 non-overlapping fields per animal (*n* = 5 per group). Scale bars = 50 µm. (**K**) Representative images of kidney TEM histology sections (left panel). Yellow arrows indicate individual podocyte foot processes. Quantitative assessment of podocyte foot-process width (FPW, right panel). Ten profiles were analyzed per animal (*n* = 3 per group). Dot plots show individual values with bars representing the mean ± SEM. * *p* < 0.05, ** *p* < 0.01, *** *p* < 0.005.

The DBA/2 mouse strain, which is more susceptible to DN than C57BL/6J [31,32], was used in this study to develop obesity-induced DN. Briefly, DBA/2 mice were fed an HFD with or without ST32da supplementation to simulate obesity-induced chronic kidney disease and chronic low-grade inflammation and assessed glomerular changes (Figure 4A). ST32da treatment improved body weight and glucose levels in DBA/2 mice (Figure 4B,C) compared with those in db/db mice (Figure 3B,C), which was evident after 4–6 weeks of treatment. Our findings also revealed that ST32da-treated db/db mice had higher triglyceride concentrations than the placebo-treated mice (Figure 3D). However, after 12 weeks of HFD feeding, DBA/2 mice showed a significant difference only in cholesterol levels compared to the normal diet-fed mice, with no significant differences in triglyceride levels (Figure 4D,E).

In addition, induction with STZ and HFD elevated triglyceride and cholesterol levels compared to induction with HFD alone. ST32da treatment selectively reduced serum triglyceride levels compared to the placebo treatment (Figure 4D). In DBA/2 mice fed an HFD alone, the levels of BUN, creatinine, and daily urinary albumin excretion did not differ significantly from those in normal diet-fed mice. However, STZ treatment increased these renal function indices. ST32da treatment ameliorated creatinine and albuminuria levels (Figure 4F,G), though not BUN (Figure 4H). Furthermore, ST32da reduced mesangial expansion and improved fibrosis in renal tubular epithelial cells in HFD/STZ mice (Figure 4I). Electron microscopy showed that ST32da markedly reduced podocyte foot process effacement (Figure 4J). Western blot analysis of kidney lysates from diabetic DBA/2 mice confirmed that ST32da strongly induced renal ATF3 expression in HFD/STZ-induced diabetic mice compared with that in vehicle-treated controls (Figure S6). Collectively, these findings indicate that systemic administration of ST32da attenuates DN progression in DBA/2 mouse models via upregulation of ATF3 in kidneys and improvement of metabolic and renal pathological features.

### 3.5. ST32da Attenuated High-Glucose–Induced Inflammatory Signaling via HDAC2-Associated Epigenetic Modulation in Rat Mesangial Cells

**Figure 5:**
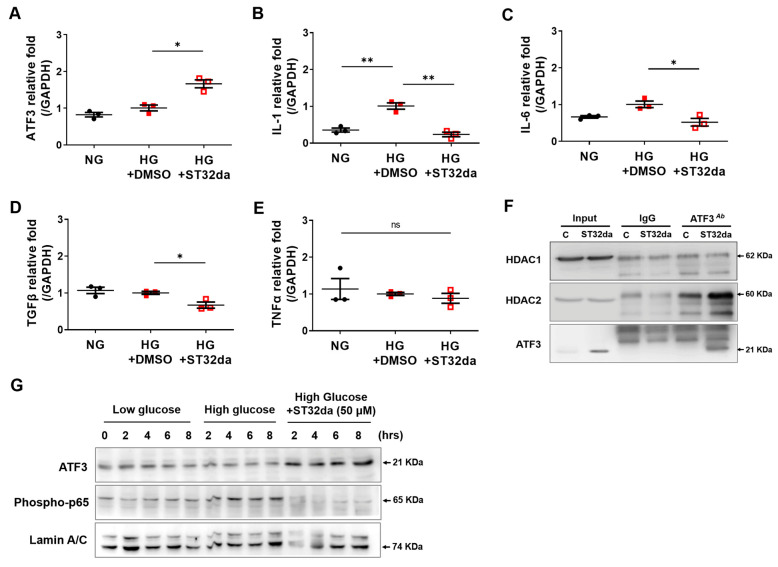
ST32da induced ATF3 expression and attenuated high-glucose inflammation in rat mesangial cells via HDAC2-dependent epigenetic modulation of NF-κB. Mesangial cell lines were pre-treated with ST32da and then incubated with either normal or high glucose concentration medium. After 24 h, cells were collected for RT-PCR assays of *Atf3* (**A**) and the pro-inflammatory cytokines *Il1* (**B**), *Il6* (**C**), *Tgfb1* (**D**), and *Tnfa* (**E**). (**F**) Increased interaction between HDAC-2 and ATF3 in the mesangial nuclear fraction when cells were treated with ST32da. (**G**) Mesangial cell lines were incubated with low or high glucose medium for 12 h and then treated with ST32da for 2, 4, 6, and 8 h. Cell lysates were probed with specific antibodies against ATF3 (cytosolic extract) and NF-κB (phospho-p65) (nuclear extract). Data were analyzed using a one-way analysis of variance followed by the post hoc Tukey’s test. Data are presented as mean ± SEM; *n* = 3 in each group. * *p* < 0.05, ** *p* < 0.01.

ATF3 interacts directly with HDAC1 and inhibits inhibit NF-κB–driven inflammation by recruiting HDAC1 to *IL6* and *IL12b* promoters, blocking transcription and reducing kidney epithelial cell apoptosis [22]. Moreover, diabetes-like conditions exacerbate glomerular podocyte cell death through high-glucose-induced mesangial hypertrophy and an inflammatory response [33]. Therefore, in this study, we used rat mesangial cell lines to examine genes related to inflammation-induced apoptosis under high glucose conditions, which are pertinent to patients with chronic kidney disease [34]. ST32da treatment induced Atf3 upregulation (Figure 5A), which in turn significantly reduced the high glucose-increased levels of IL-1, IL-6, TGFβ, and TNFα (Figure 5B–E), indicating its ability to inhibit pro-inflammatory effects in rat mesangial cells. Immunoprecipitation experiments showed that ST32da treatment increased HDAC-2 levels but not those of HDAC-1 in mesangial cells (Figure 5F). Additionally, in high-glucose conditions mimicking in vivo diabetes, mesangial cells exhibited increased nuclear translocation of NF-κB (phosphorylated p65), which was reduced by ST32da-induced Atf3 expression (Figure 5G). These findings suggest that ST32da exerts a protective role against obesity-induced DN through anti-inflammatory actions. ATF3 recruitment associated with HDAC2, antagonized NF-κB-dependent IL-6 induction in renal mesangial cells (Figure 5C,F,G).

### 3.6. IL-6 Induced Apoptosis of Cultured Podocytes

**Figure 6:**
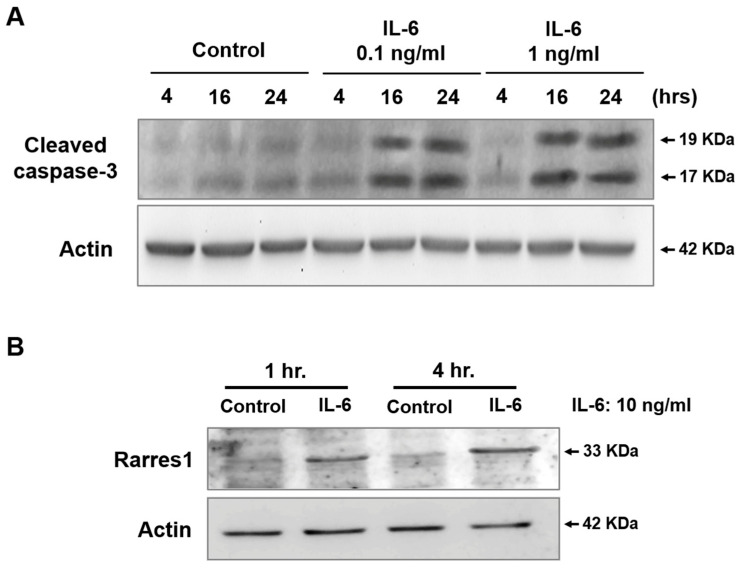
IL-6 induced RARRES1 and caspase-3 activation in podocytes. (**A**) Renal podocytes (MCP-5 cells) were treated with 0.1 ng/mL or 1 ng/mL of IL-6 for different periods, and cell lysates were probed for the active form of caspase-3 and (**B**) RARRES-1 levels. Anti-actin served as a loading control for the cytosolic or nuclear fraction, respectively. Experiments were performed in two replicates.

Chronic kidney disease incidence correlates with elevated systemic IL-6 levels [35]. Additionally, RARRES1 expression in glomeruli, predominantly in podocytes, is elevated in patients with diabetic kidney disease [12]. Because ST32da suppresses IL-6/NF-κB signaling in mesangial cells (Figure 5), we next asked whether IL-6 is sufficient to drive podocyte apoptosis via RARRES1. Exposure of mouse podocytes to IL-6 resulted in a time-dependent increase in cleaved caspase-3 at 16–24 h (Figure 6A) and a rapid induction of RARRES1 expression at 4 h (Figure 6B), as determined by Western blot analysis. These findings suggest that IL-6 contributes to podocyte apoptosis by upregulating RARRES1.

**Figure 7:**
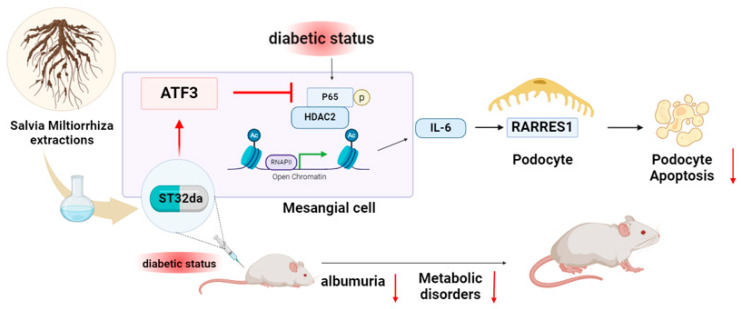
Proposed mechanism of ST32da-mediated renoprotection in diabetic nephropathy.

Integrating our findings, we propose a working model for the action of ST32da. During DN progression, patients with T2DM exhibit reduced renal ATF3 expression. Pharmacologic induction of ATF3 by ST32da restores ATF3 levels and facilitates the recruitment of HDAC2 to the NF-κB complex, resulting in histone deacetylation and transcriptional repression of IL-6. The consequent decrease in mesangial IL-6 secretion mitigates paracrine activation of RARRES1 in podocytes, thereby reducing caspase-3 activation and podocyte apoptosis. In vivo, this mechanism aligns with the observed attenuation of albuminuria, preservation of podocyte structure, and improvement of metabolic profiles in diabetic mouse models treated with ST32da. Collectively, these results delineate a model in which ST32da acts upstream at the ATF3–HDAC2–NF-κB axis to suppress IL-6–mediated mesangial–podocyte signaling and ameliorate diabetic kidney injury (Figure 7).

## 4. Discussion

Identifying pharmacologic interventions that target both obesity and its metabolic complications is of clinical urgency owing to the increased incidence of chronic diseases relating to obesity. In this study, we demonstrated the potency of ST32da, an ATF3 inducer derived from *Salvia miltiorrhiza*, as a novel class of drugs for obesity-related metabolic disorders, including obesity-related DN. ST32da has previously been shown to restore the lipolysis/lipogenesis balance, increase functional brown adipose tissue without suppressing dietary intake, and ameliorate diabetes [21]. Here, we demonstrated that Atf3 loss exacerbated HFD/STZ-induced obesity-related DN, reduced body weight, and increased mortality and glomerular fibrosis in vivo (Figure S7). Using various obesity-related DN mouse models, we showed that ST32da-induced ATF3 benefits obesity-related DN and improves high-glucose-induced inflammatory reactions in mesangial cells by inhibiting the proapoptotic molecule RARRES1, activated via the paracrine pathway. These findings suggest that ATF3 inducers could be promising candidates for treating and preventing obesity-related DN.

ST32da was initially designed by referring to its basic structure from *Salvia miltiorrhiza* extraction, known for its cardiovascular benefits and platelet aggregation inhibitory effects [36]. In a DBA mouse model of obesity-related DN, treatment with the liposoluble extract of *Salvia miltiorrhiza* improved the glomerular filtration rate and renal function parameters (data not shown). More than 30 liposoluble components have been isolated from *Salvia miltiorrhiza*, primarily conjugated quinones and ketones, including tanshinone I, tanshinone IIA, and cryptotanshinone [37]. Structurally, ST32da resembles tanshinone I and tanshinone IIA, differing mainly in conjugated side chains. To investigate potential mechanistic links, we examined the renal protective effects of tanshinone IIA, which has previously been shown to induce ATF3 expression [38]. However, tanshinone IIA, the primary component of *Salvia miltiorrhiza*, did not exhibit protective effects in the chronic kidney disease 5/6 NxBL mouse model (data not shown). These findings suggested that the kidney-protective components of *Salvia miltiorrhiza* might differ from those of tanshinone IIA. The variation in conjugated side chains likely influences signaling pathways, modulating ATF3 expression and ultimately contributing to varying degrees of renal protection. Furthermore, the structural isomerism between ST32da (trans form) and ST32db (cis form) may account for their differential activity, as evidenced by significantly different effective doses in HFD-induced obesity models (10 mg/kg/week for ST32da [21] vs. 75 mg/kg/week for ST32db [38]). Notably, ST32db ameliorates HFD-induced obesity and metabolic dysfunction via ATF3-dependent suppression of C/EBPα-driven adipogenic programs [38]. These findings position ATF3 induction as a shared upstream mechanism across this chemical family and provide a metabolic context for the renoprotective effects observed here with ST32da under diabetic conditions. To further explore the mechanisms by which ST32da or ST32db induces ATF3, we conjugated ST32da with biotin for immunofluorescence analysis. Treatment with this biotinylated compound increased ATF3 activity in treated cells, and fluorescence was observed only in the cytoplasm within 30 min, indicating that lipid-soluble ST32da directly passes through the cell membrane and enters the cytoplasm. However, future studies should employ proteomic mass spectrometry analysis of immunoprecipitates from with ST32da-biotin-treated cells to identify ST32da-associated proteins contributing to the increased expression of ATF3.

The term “fatty kidney” has recently been employed to represent the pathological conditions of lipid deposition in the kidneys, mirroring the term “fatty liver” [39]. Fatty kidneys exhibit common traits due to the harmful effects of lipids, termed renal lipotoxicity [40]. Lipotoxicity, from ectopic lipid accumulation in the kidney, leads to inflammatory cell recruitment, oxidative stress, fibrosis, and ER stress [41]. Moreover, obesity-induced liver steatosis, causing kidney lipotoxicity, aligns with a subclinical inflammatory state that promotes pro-inflammatory factors implicated in insulin resistance [42] or obesity-related kidney diseases such as DN [43]. These adverse effects can be reversed by peroxisome proliferator-activated receptor-gamma coactivator alpha (PGC1α) or peroxisome proliferator-activated receptor gamma agonists, enhancing renal function during obesity [44,45]. In anti-obesity studies, Atf3^−/−^ mice exhibited severe liver lipid deposition compared to WT mice [21]. In our previous study, both i.p. and oral administration of ST32da reduced hepatic lipid deposition via PGC1α expression, lowering serum triglyceride levels, liver weight, and GPT levels by increasing ATF3 expression [21]. In the current study, ST32da treatment significantly reduced tubular injury in HFD-induced DN mice, particularly in the proximal convoluted tubule (Figure S8). These findings indicate that ST32da may confer renal anti-lipotoxicity in DBA mice, but not in db/db mice, which may involve reducing hepatic steatosis or triglyceride levels by activating the PGC-1α pathway and increasing ATF3 expression, thereby inhibiting pro-inflammatory factors such as IL-6, IL-1, and TGF-β (Figure 5B–D), ultimately improving obesity-related DN. Recent studies indicate that adipokines, lipokines, or microRNAs secreted from brown adipose tissue can protect kidney function in diabetes [46,47], involving the activation of the renal AMP-activated protein kinase/sirtuin 1/PGC1α signaling pathway. Our previous study demonstrated that ST32da promotes ATF3 expression, inducing adipocyte browning by suppressing carbohydrate-responsive element-binding protein stearoyl-CoA desaturase-1 and enhancing the PGC-1 axis in HFD-induced obese mice [21]. Thus, the protective effect of ST32da on DN might involve increasing adipokines such as FGF21 or neuregulin 4. However, further mass spectrophotometry analysis in DN mouse models is necessary.

Obesity-related DN is closely associated with chronic, low-grade inflammation [48,49] and cytokine expression (IL-1α, IL-6, TGFβ, and TNFα), leading to leukocyte recruitment and DN [49]. Studies have identified ATF3 as a negative regulator of inflammation in the kidney and other organs [22,27,50]. Zhang et al. found inducible ATF3 expression in podocytes of patients with DN or db/db mice, with minimal positive expression levels observed in immunofluorescence experiments. ATF3 is primarily located in mesangial cells within glomeruli, not in podocytes [28]. Xu et al. showed the involvement of ATF3 in glomerular mesangial cell apoptosis following a sublytic C5b-9 attack [51]. We speculate that the aggravation of diabetic proteinuria in Atf3^−/−^ mice results from low-grade inflammation; this is consistent with previous studies indicating mesangial cells regulate in situ immunity via paracrine cytokines and promote immune cell infiltration, worsening kidney function [52,53,54]. The inhibitory effect of ATF3 on IL-1/IL-6 or TGFβ reduces cytokine production and podocyte apoptosis, implicating the role of these cytokines. Previous studies on ATF3 have shown its ability to regulate the translocation of NF-κB p65, leading to reduced production of IL-6 and TNFα [22,55]. The variations in cytokine expression induced by ATF3 through ST32da may be related to ATF3 binding to different proteins, such as HDAC1/2, Jun, Fos, and P300, thereby altering chromatin structure and restricting transcription factor access [56]. The HDAC family, associated with ATF3, participates in anti-inflammatory effects in the kidney through HDAC1 [22,57], silencing ER stress via HDAC1/3 [58] or metabolic reprogramming via HDAC1/2. This suggests that different cell types or organs may determine the specific HDAC type that combines with ATF3. In the current study, ATF3 was bound to HDAC2 in mesangial cells but not to HDAC1 in renal epithelial cells. TGFβ has been shown to promote cell proliferation and extracellular matrix deposition in mesangial cells [59]. Overexpression of ATF3 by ST32da can inhibit the TGFβ/IL-6 axis (Figure S9), and in vitro studies of cultured glomerular mesangial cells show ATF3 can simultaneously inhibit factors such as profibrotic TGF-β and inflammatory cytokines IL-6 and TNF-α, which promote cell proliferation and extracellular matrix deposition.

Although our findings indicate that ST32da holds promise for ameliorating diabetic nephropathy, several limitations warrant consideration to contextualize the results and guide future research. First, although multiple mouse strains (C57BL/6, Atf3^−/−^, DBA/2, and db/db mice) were used to model obesity-related diabetic nephropathy, inherent species-specific differences in renal physiology and disease trajectories limit direct extrapolation to humans. Notably, db/db mice develop only modest albuminuria and mesangial expansion without advanced glomerulosclerosis or tubulointerstitial fibrosis, thereby representing early rather than progressive stages of human diabetic nephropathy [31]. Second, our investigation focused exclusively on the direct protective mechanisms of ST32da in renal cells, without exploring its systemic effects or inter-organ crosstalk. Visceral adipose tissue has long been implicated in the pathogenesis of chronic kidney disease [60], with emerging evidence highlighting perirenal fat as a potentially more direct contributor to renal functional decline, proteinuria, and structural alterations compared to general visceral adiposity [61]. Our prior studies demonstrated that ST32da promotes brown adipose tissue expansion while reducing white adipose tissue accumulation [21]. Thus, it is plausible that the observed renal benefits may partially stem from attenuated inflammation in white adipose tissue rather than solely from direct renal protection, underscoring the need for comprehensive analyses of adipose-renal interactions in future work. Third, although in our previous and the present studies, ST32da did not exhibit hepatic or renal toxicity in mice following 12–16 weeks of oral or intraperitoneal administration at therapeutic dose [21], comprehensive toxicologic profiling was not performed. Given that a structurally related isomer (ST32dc) demonstrated hepatotoxicity in previous studies [38], future work should define the minimum lethal dose (LD_50_), and potential off-target effects of ST32da to support translational development.

In conclusion, this study identified ST32da as a novel anti-obesity drug that leverages the anti-inflammatory effect of ATF3 to protect against DN and kidney lipotoxicity. Our findings emphasize the importance of ATF3-mediated HDAC2 recruitment by ST32da in the epigenetic regulation of NF-κB-induced IL-6 expression in mesangial cells, ultimately reducing podocyte apoptosis under high glucose stress. Therefore, ATF3 overexpression via ST32da or pharmacological HDAC2 modulation may be an effective strategy for preventing chronic renal injury, positioning ST32da as a potential future clinical drug.

## Figures and Tables

**Table 1:** Primer sequences for Real-time PCR.

Gene Name	Forward Primer 5′-3′	Reverse Primer 5′-3′
ATF3	AgAgTgTgAATgCCgAACTg	ggATAAAAAggTTCCTCTCgTC
IL1α	AAAgCTgCTTCgTTAAATgACC	TgAgCACTCACAAAgAgCTgAg
IL6	ATTgTATgAACAgCgATgATgC	AggTAgAAACggAACTCCAgAAg
TGFβ1	CCTgAgTggCTgTCTTTTgA	CgTggAgTACATTATCTTTgCTg
TNFα	AgACCCTCACACTCAgATCA	gTCTTTgAgATCCATgCCATTg
GAPDH	CATTCTTCCACCTTTgAT	CTgTAgCCATATTCATTgT

## Data Availability

The data to support the findings of the present study are available from the corresponding author upon reasonable request.

## Supplementary Materials

The following supporting information can be downloaded at https://www.mdpi.com/article/10.3390/cells14231893/s1, Figure S1: Synthetic route to desired compound ST32da (compound 6)* and ST32db (compound 6′)*. Figure S2: 1H-NMR spectrum of compound ST32da. Figure S3: 13C-NMR spectrum of compound ST32da. Figure S4: ATF3^−/−^ Mice Exhibit Complete Loss of ATF3 mRNA and Protein in Renal Tissue. Figure S5: ST32da induces ATF3 expression in HK-2 cells in a dose-dependent manner. Figure S6: ST32da enhances renal ATF3 protein expression in diabetic DBA/2 mice. Figure S7: Loss of ATF3 in mice aggravated high-fat diet (HFD)-induced obesity and metabolic dysfunction. Figure S8: Representative photographs of PAS staining in DBA mice with or without treatment with ST32da under a high-fat diet-induced diabetic nephropathy model. Figure S9: Effect of Atf3 inducer, ST32da, on Atf3 and *Il6* expression in the mesangial cells after TGF-β1 treatment. Table S1: Interpretation of ^13^C NMR and ^1^H NMR data of ST32da.

## Abbreviations

ATF3	Activating transcription factor 3
BUN	Blood urea nitrogen
cDNA	Complementary DNA
db/db	Diabetes (leptin receptor–deficient) mouse strain
DN	Diabetic nephropathy
HFD	High-fat diet
HG	High glucose
HDAC1/2	Histone deacetylase 1/2
IL-1α	Interleukin-1 alpha
IL-6	Interleukin-6
i.p.	Intraperitoneal
MCP-5	Mouse podocyte clone 5
NF-κB	Nuclear factor kappa-light-chain-enhancer of activated B cells
NMR	Nuclear magnetic resonance
PAS	Periodic acid–Schiff
PCR/RT-qPCR	Polymerase chain reaction/Real-time quantitative PCR
PGC-1α	Peroxisome proliferator-activated receptor gamma coactivator-1 alpha
RARRES1	Retinoic acid receptor responder protein 1
RNA	Ribonucleic acid
RPMI	Roswell Park Memorial Institute medium
RT-qPCR	Real-time quantitative PCR
SEM	Standard error of the mean
SDS-PAGE	Sodium dodecyl sulfate polyacrylamide gel electrophoresis
STZ	Streptozotocin
T2DM	Type 2 diabetes mellitus
TEM	Transmission electron microscopy
TGF-β/TGF-β1	Transforming growth factor beta/beta-1
TNF-α	Tumor necrosis factor alpha
WT	Wild type
