# Supplementary material for: ST32da, a Novel Salvia miltiorrhiza-Derived ATF3 Inducer, Alleviates Obesity-Related Diabetic Nephropathy in Mouse Models
Source: Cells. 2025 Nov 28;14(23):1893. doi: 10.3390/cells14231893 (PMC12691026; doi:10.3390/cells14231893)
Supplement: Supplementary file 1 [file cells-14-01893-s001.zip › cells-3984244-supplementary.pdf]

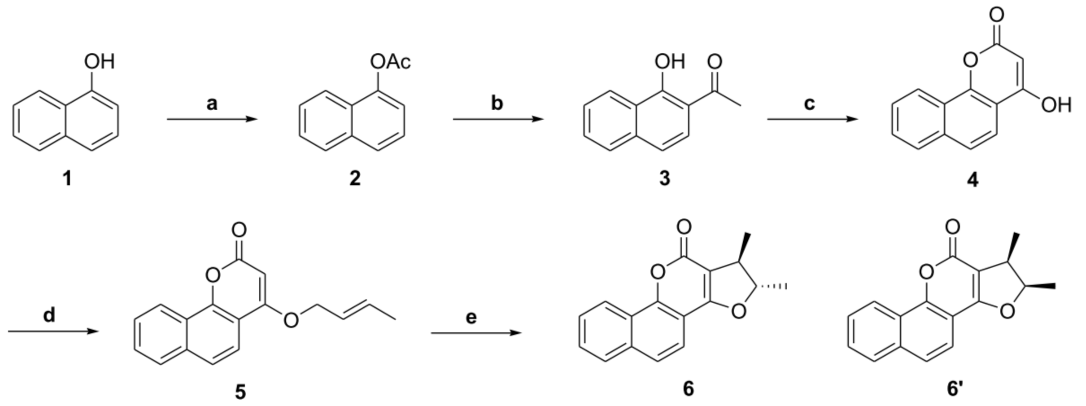

Reagents and conditions: a) acetic anhydride, DMAP, pyridine, reflux; **b**) $AlCl_3$, 135°C; **c**) diethyl carbonate, NaH, toluene, 120°C; **d**) crotyl bromide, K_2CO_3, acetone, 60°C; **e**) BF_3-Et_2O, DMF, 140°C, M.W..

Figure S1. Synthetic route to desired compound ST32da (compound 6)* and ST32db (compound 6')*

*The residue was purified by flash chromatography (silica gel; EtOAc/n-hexane, 1:10) to give ST32da and ST32db as light-yellow solid

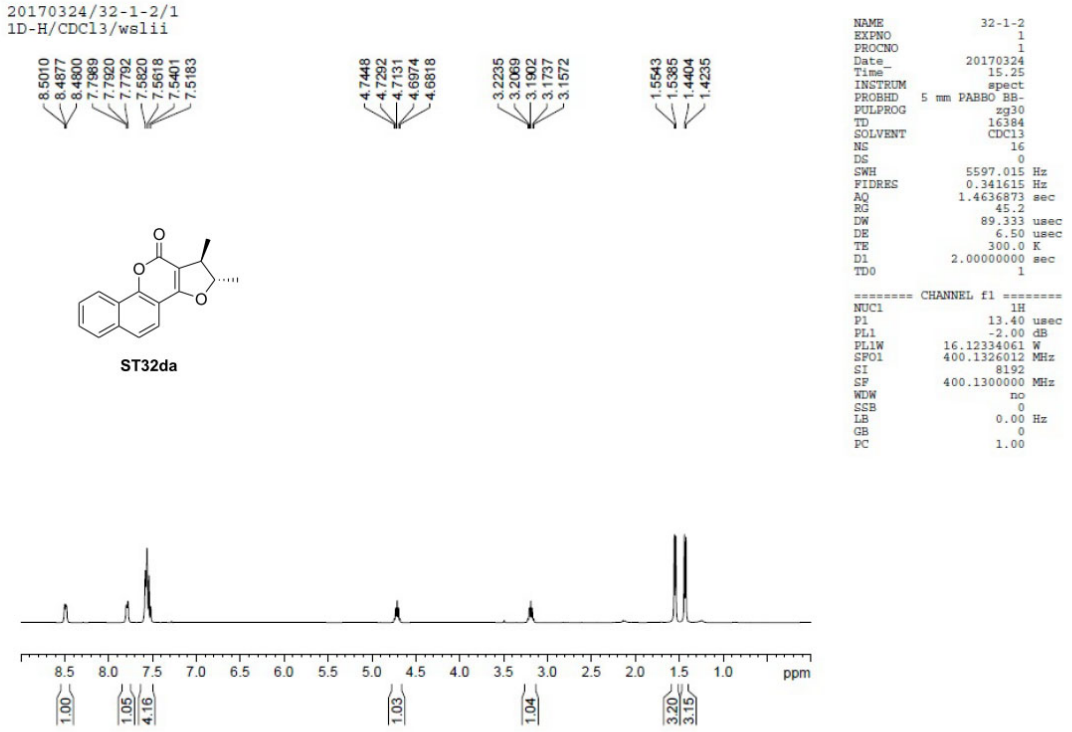

Figure S2. ^{1}H-NMR spectrum of compound **ST32da** ($CDCl_3$, 400MHz)

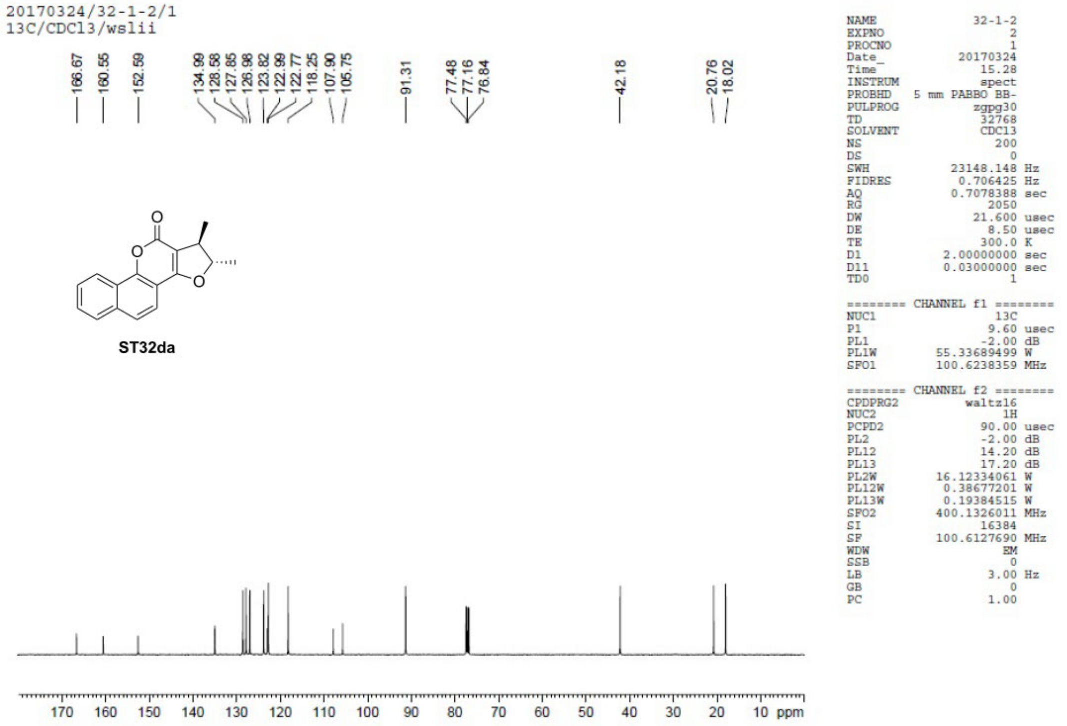

Figure S3. ^{13}C-NMR spectrum of compound **ST32da** ($CDCl_3$, 100MHz)

Table S1 Interpretation of ^{13}C NMR and ^{1}H NMR data of ST32da

(δ in ppm, *J* in Hz, in $CDCl_3$, 400 MHz)

position	δ_C	Type	δ_H	Multiplicity
1	166.7	C	—	—
2	160.5	C	—	—
3	152.6	C	—	—
4	135.0	C	—	—
5	128.6	CH	7.79	d
6	127.9	CH	7.55	dd
7	127.0	CH	7.55	dd
8	123.8	CH	7.55	d
9	123.0	CH	7.55	d
10	122.8	CH	8.50	d
11	118.2	C	—	—
12	107.9	C	—	—
13	105.7	C	—	—
14	91.3	CH	4.71	dq
15	42.2	CH	3.19	dq
16	20.8	CH_3	1.55	d
17	18.0	CH_3	1.43	d

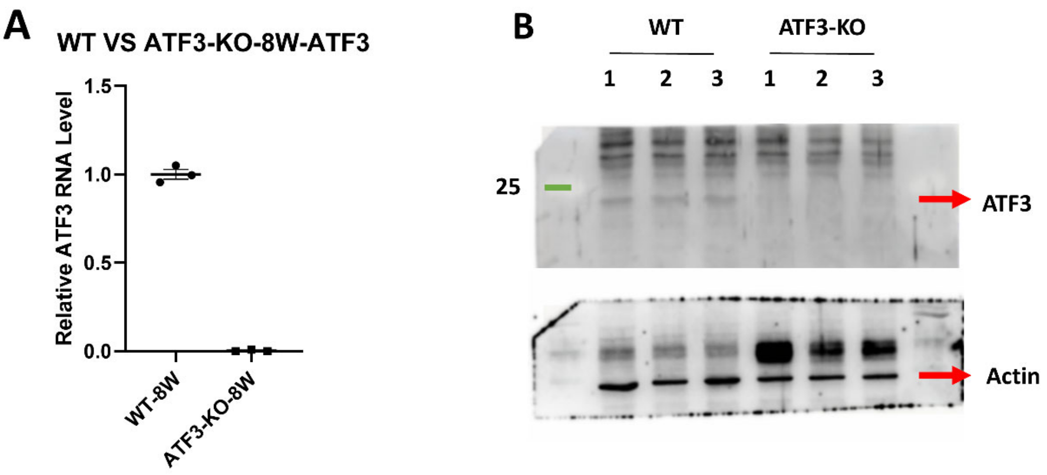

Figure S4 ATF3-KO Mice Exhibit Complete Loss of ATF3 mRNA and Protein in Renal Tissue. (A) **Quantification of renal *Atf3* mRNA in 8-week-old WT and *Atf3*$^{-/-}$ mice.** Relative *Atf3* transcript levels were measured by quantitative real-time PCR in tissue samples from *Atf3* gene-deleted mice (*Atf3*$^{-/-}$) and their wild-type littermates (WT) at 8 weeks of age. Expression in each sample was normalized to an internal reference gene and is presented relative to the mean of the WT group (set to 1.0). Individual data points represent biological replicates (n = 3 per group), and horizontal bars indicate the group mean ± SEM. *Atf3*$^{-/-}$ mice display complete loss of ATF3 mRNA expression compared to WT controls.

(B) **Western blot analysis of renal ATF3 protein expression in 8-week-old WT and *Atf3*$^{-/-}$ mice.** Red arrows indicate ATF3 (~25 kDa; green tick) in the top panel and β-actin in the bottom panel. WT mice lanes 1–3 show ATF3 expression, whereas *Atf3*$^{-/-}$ mice lanes 1–3 lack the ATF3 band; actin is uniform across all lanes.

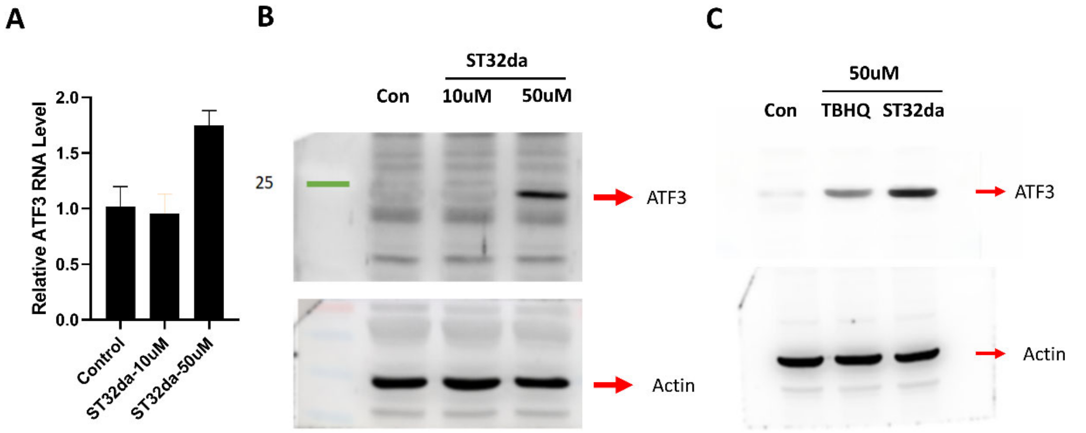

Figure S5. ST32da induces ATF3 expression in HK-2 cells in a dose-dependent manner.

(A) Quantitative RT-PCR analysis of *Atf3* mRNA levels in HK-2 human proximal tubular epithelial cells following 24-hour treatment with vehicle control, 10 µM ST32da, or 50 µM ST32da. Transcript levels were normalized to an internal reference gene and expressed relative to the mean value of the vehicle-treated group (set to 1.0). Data represent the mean ± SEM from three independent experiments.

(B) Representative immunoblot demonstrating ATF3 expression following ST32da treatment. Total cellular proteins were extracted after 24 h exposure to vehicle (Control) or ST32da (10 µM and 50 µM). Blots were probed with an anti-ATF3 antibody; β-actin served as the loading control. A clear dose-responsive elevation of ATF3 was detected, reaching robust significance only at 50 µM ST32da, whereas 10 µM did not appreciably alter ATF3 abundance.

(C) Representative Western blot illustrating the effect of ST32da on ATF3 protein expression. HK-2 human proximal tubular epithelial cells were treated for 24 h with vehicle (Con), tert-butylhydroquinone (TBHQ, 50 µM; positive control), or ST32da (50 µM). Membranes were sequentially probed with antibodies against ATF3 and β-actin, the latter serving as the loading control. TBHQ was included as a canonical inducer of ATF3.

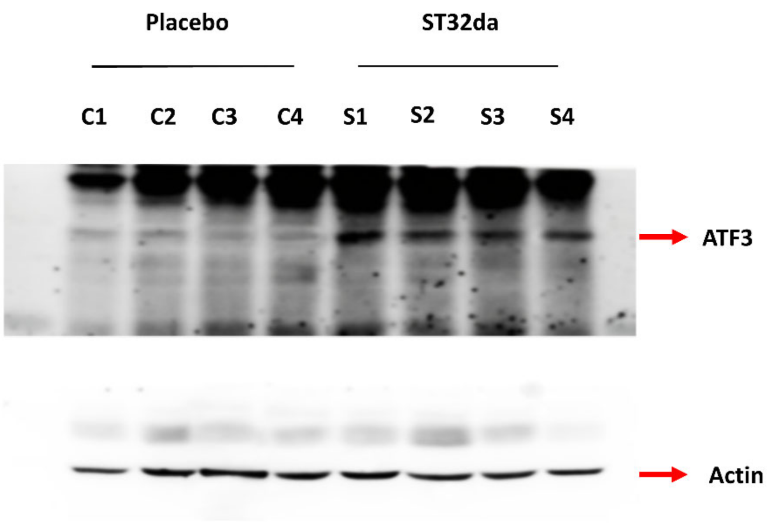

Figure S6. ST32da enhances renal ATF3 protein expression in diabetic DBA/2 mice.

Representative Western blot analysis of kidney lysates collected from high-fat diet/streptozotocin (HFD/STZ)–induced diabetic DBA/2 mice that received either vehicle (Placebo; lanes C1 – C4) or ST32da (5 mg/kg twice per week; lanes S1 – S4) for 6 weeks. Each lane corresponds to an individual mouse. β-actin was probed on the same membrane as a loading control. The noticeably stronger ATF3 signal in ST32da-treated samples (S1 – S4) compared with placebo controls demonstrates that ST32da administration up-regulates ATF3 protein abundance in vivo.

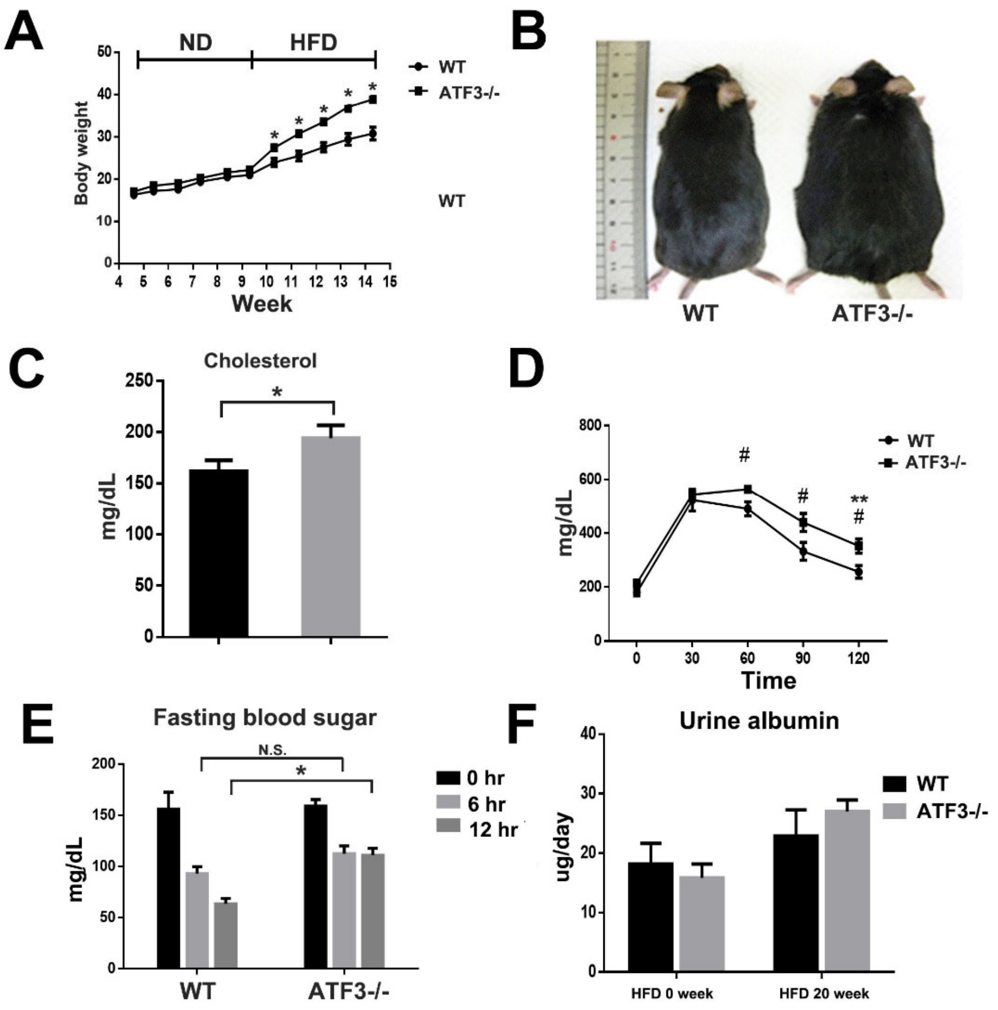

Figure S7. Loss of ATF3 in mice aggravated high-fat diet (HFD)-induced obesity and metabolic dysfunction. *Atf3* gene-deleted mice (*Atf3*$^{-/-}$) and their wild-type littermates (WT) were fed a normal diet (ND) or an HFD for five or six weeks, respectively. Measurements were performed after six weeks of HFD feeding. (A) Body weight of WT and *Atf3*$^{-/-}$ mice fed an ND or HFD, and (B) body images. (C) Serum cholesterol levels. (D) Glucose tolerance tests. (E) Serum glucose levels. (F) Urine albumin levels. For (A), n = 8 per group; wild-type and *Atf3*$^{-/-}$. Data are presented as mean ± SEM and *p < 0.05 vs. WT.

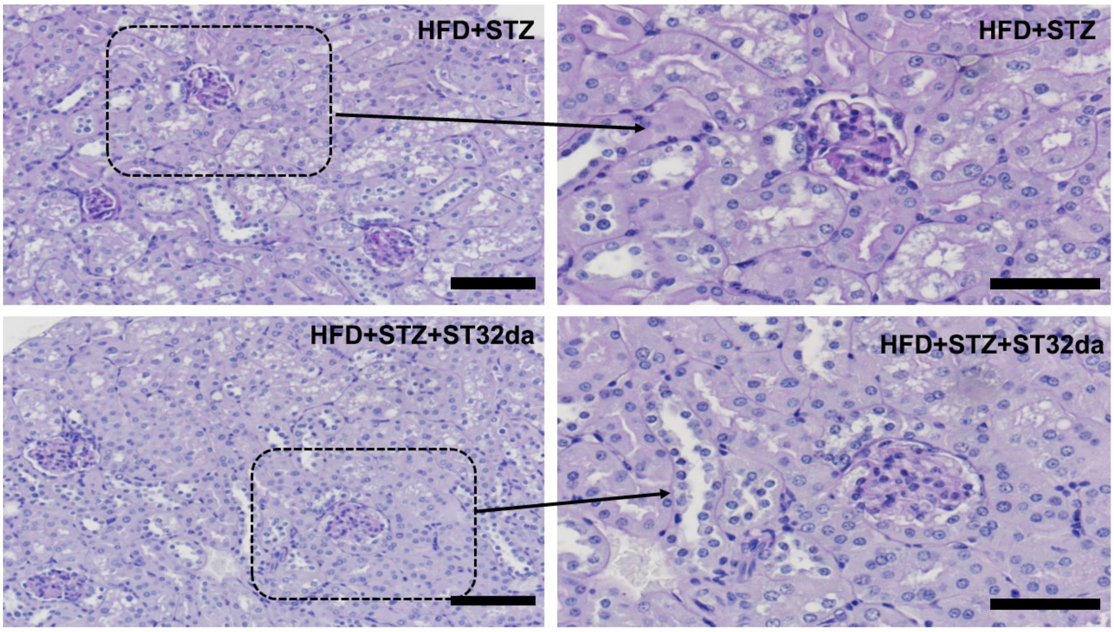

Figure S8. Representative photographs of PAS staining in DBA mice with or without treatment with ST32da under a high-fat diet-induced diabetic nephropathy model.

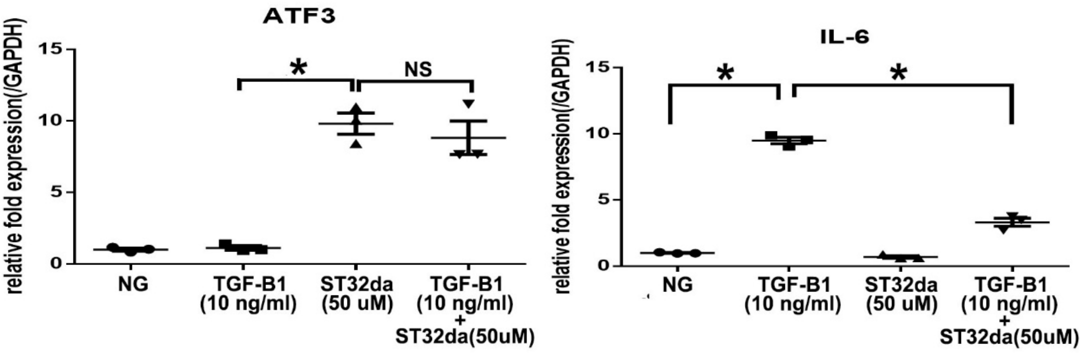

Figure S9. Effect of *Atf3* inducer, ST32da, on *Atf3* and *Il6* expression in the mesangial cells after TGF-β1 treatment. The cells were treated with ST32da and TGF-β1 simultaneously for 8 h. RNA was purified from cell lysates and analyzed by qPCR using *Atf3*- and *Il6*-specific primers.
